# A Comparison of Gene Region Simulation Methods

**DOI:** 10.1371/journal.pone.0040925

**Published:** 2012-07-18

**Authors:** Audrey E. Hendricks, Josée Dupuis, Mayetri Gupta, Mark W. Logue, Kathryn L. Lunetta

**Affiliations:** 1 Department of Biostatistics, Boston University School of Public Health, Boston, Massachusetts, United States of America; 2 Bioinformatics Program, Boston University, Boston, Massachusetts, United States of America; 3 Department of Biomedical Genetics, Boston University School of Medicine, Boston, Massachusetts, United States of America; Aarhus University, Denmark

## Abstract

**Background:**

Accurately modeling LD in simulations is essential to correctly evaluate new and existing association methods. At present, there has been minimal research comparing the quality of existing gene region simulation methods to produce LD structures similar to an existing gene region. Here we compare the ability of three approaches to accurately simulate the LD within a gene region: HapSim (2005), Hapgen (2009), and a minor extension to simple haplotype resampling.

**Methodology/Principal Findings:**

In order to observe the variation and bias for each method, we compare the simulated pairwise LD measures and minor allele frequencies to the original HapMap data in an extensive simulation study. When possible, we also evaluate the effects of changing parameters.

HapSim produces samples of haplotypes with lower LD, on average, compared to the original haplotype set while both our resampling method and Hapgen do not introduce this bias. The variation introduced across the replicates by our resampling method is quite small and may not provide enough sampling variability to make a generalizable simulation study.

**Conclusion:**

We recommend using Hapgen to simulate replicate haplotypes from a gene region. Hapgen produces moderate sampling variation between the replicates while retaining the overall unique LD structure of the gene region.

## Introduction

Many new statistical methods and algorithms to detect association between a trait and one or more genetic variants have recently been developed to analyze the abundance of data produced by Genome Wide Association Studies (GWAS). Simulated data are used to verify and compare the type-I error rates and power of these new association methods. The methods are often compared over a variety of gene region, phenotypic, and association simulation scenarios [1], [2], [3], [4]. The foundation of genetic association studies, as well as the basis for most new complex analyses that use GWAS data, presumes the ability to detect associated genetic variants through linkage disequilibrium (LD) with genotyped markers. However, association methods incorporate, control, or exploit the regional LD to various extents and using different strategies. Thus, accurately modeling LD in simulations is essential to correctly evaluate and compare new association methods. Despite the need, there currently exists no comprehensive comparison of the most promising gene region simulation methods. Here, we compare three approaches on their ability to accurately simulate the LD within a gene region: resampling, Hapgen [5], [6], and HapSim [7]. In addition, we assess the impact of various parameters, such as recombination levels and mutation rates, on the simulated LD structure. In order to compare the variation and bias of the simulation replicates to the original HapMap data, we evaluate the pairwise LD measures and marker descriptive statistics for each method over 100 simulation replicates.

Genetic data simulation was first developed within population genetics theory. Methods developed from population genetics theory, called forward time and backwards time (or coalescent) methods, often simulate haplotypes without relying on real data, and instead only use parameters to model aspects of population genetics such as recombination, gene conversion, and evolutionary models. More recently, researchers have developed methods that simulate directly from an existing sample of haplotypes. We describe these methods further below.

Simulating directly from a set of existing haplotypes avoids relying exclusively on subjective parameters and is likely to give a representative picture of the complex underlying LD structure in a gene region since the methods start with real data. Further, simulating directly from a gene region is relatively straightforward and is computationally efficient. Therefore, in this paper we focus on methods that simulate from a set of observed haplotypes in a gene region.

In addition to focusing on gene region simulation methods, we further concentrate on methods that appear to or claim to be able to simulate pairwise LD similar to the original sample of haplotypes over at least a 100 Kb chromosomal region, and can take any set of haplotypes as a starting sample. Three methods that meet this criteria are Hapgen [5], [6], HapSim [7], and resampling haplotypes.

In 2003, Li and Stephens used an approximation to conditional probability to relate a distribution of haplotypes to a recombination rate that varies across a chromosomal region [5]. While in their initial paper Li and Stephens focused on identifying recombination hotspots using a set of haplotypes, a clear extension of this method is to simulate haplotypes given an initial sample of haplotypes and recombination rates across the region. Using an existing set of phased haplotypes, the extension of Li and Stephen’s method [6] uses a Hidden Markov Model to create new haplotypes that are mosaics of the original set. This simulation method was implemented as a software, Hapgen, that further extends the approach to incorporate point mutations in addition to a variable recombination rate [6].

**Figure 1:**
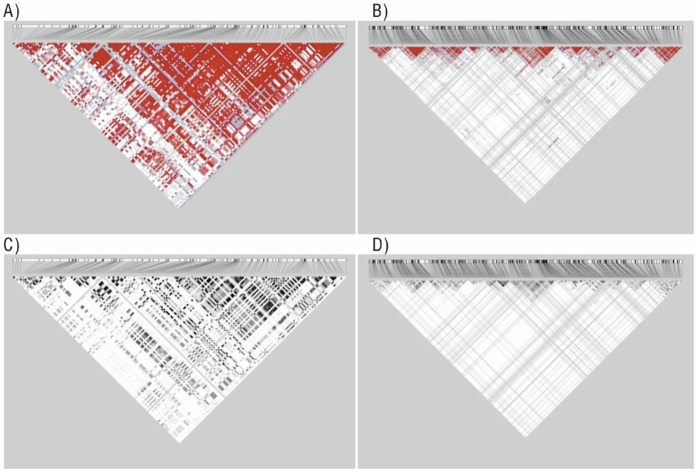
Gene Region LD plots. A) D’ Gene Region 1 (300 Kb), B) D’ Gene Region 2 (1000 Kb), C) r^2^ Gene Region 1 (300 Kb), D) r^2^ Gene Region 2 (1000 Kb).

**Table 1:** MAF–Gene Region 1*.

Method	N	Min	Q1	Median	Q3	Max	Mean	SD
**Hapgen**	14500	−0.074	−0.010	−0.001	0.009	0.068	−0.001	0.017
**Resampling**	14500	−0.047	−0.005	<0.001	0.004	0.033	<0.001	0.008
**HapSim**	14300**	−0.042	−0.005	<0.001	0.004	0.038	<0.001	0.008

* Change in MAF from original HapMap MAF for each pair of SNPs in Gene.

Region 1 (MAF_simulated_ – MAF_HapMap_).

** HapSim requires that all monoallelic SNPs are removed prior to simulation.

Resampling haplotypes is probably the most straight forward simulation method. It was first described by de Bakker et al. in a paper looking into the efficiency and power of GWAS [3]. This method samples with replacement from a set of existing haplotypes to create simulated replicates. We implement a minor extension of resampling haplotypes that recombines two of the original haplotypes chosen at random using a recombination rate, which is allowed to vary across the region. This method may be thought of as producing a sample of haplotypes similar to that seen by a single generation of random mating. Conversely, Hapgen creates new haplotypes that are mosaics of all original and newly created haplotypes, which may be thought of as a sample of haplotypes similar to several generations of random mating. Due to this difference, we may expect Hapgen to introduce more variation than our extension of haplotype resampling.

In HapSim [7], the program simulates vectors from a multivariate normal distribution using a correlation matrix estimated from the minor allele frequency (MAF) and joint probabilities of the original set of markers. HapSim then assigns a 0 or 1 for each variable along the vector using a cutoff defined by the MAF estimated from the original sample. Unlike Hapgen, HapSim’s parameters are not adjustable as they are ingrained within the choice of simulating from a multivariate normal distribution. Thus, this approach is less subjective but is also less modifiable.

Before comparing methods, it is important to establish the desired characteristics of the simulation replicates. As with any sample of simulated replicates, there should be some variation. We believe that too little variation limits the generalizability of the simulation study while too much variation may be unrealistic and might break down the characteristics of the gene region used for simulation. Thus, the ideal simulation method will produce replicates that differ enough to produce sampling variability but not so much that the unique characteristics of the particular gene region are lost. However, the ideal amount of variation is difficult to quantify. Further, we believe that when simulation is used to evaluate association analysis methods for a specific gene region, a desirable characteristic is that the method produces replicates that do not, on average, introduce an overall loss or gain in LD. This is the main characteristic that we evaluate in this article. We also examine the variation and potential bias of MAF.

Several previous reviews of haplotype simulation methods as well as papers describing a new method exist [7], [8], [9], [10], [11]. However, the reviews do not focus only on methods that simulate from an existing region, and, more critically, do not provide parallel comparison and implementation of the methods [8], [9], [11]. Further, papers that describe a particular method [7], [10], [12] often provide results only for a single replicate, and usually do not compare the simulation method to other available methods.

Here, we compare, through parallel implementation, the ability of Hapgen, HapSim, and resampling to simulate a gene region without introducing an overall loss or gain in LD across the region.

**Table 2:** MAF–Gene Region 2*.

Method	N	Min	Q1	Median	Q3	Max	Mean	SD
**Hapgen**	44500	−0.086	−0.010	<0.001	0.009	0.093	<0.001	0.018
**Resampling**	44500	−0.039	−0.004	<0.001	0.004	0.043	<0.001	0.008
**HapSim**	37900**	−0.041	−0.005	<0.001	0.005	0.037	<0.001	0.008

* Change in MAF from original HapMap MAF for each pair of SNPs in Gene.

Region 2 (MAF_simulated_ – MAF_HapMap_).

** HapSim requires that all monoallelic SNPs are removed prior to simulation.

**Figure 2:**
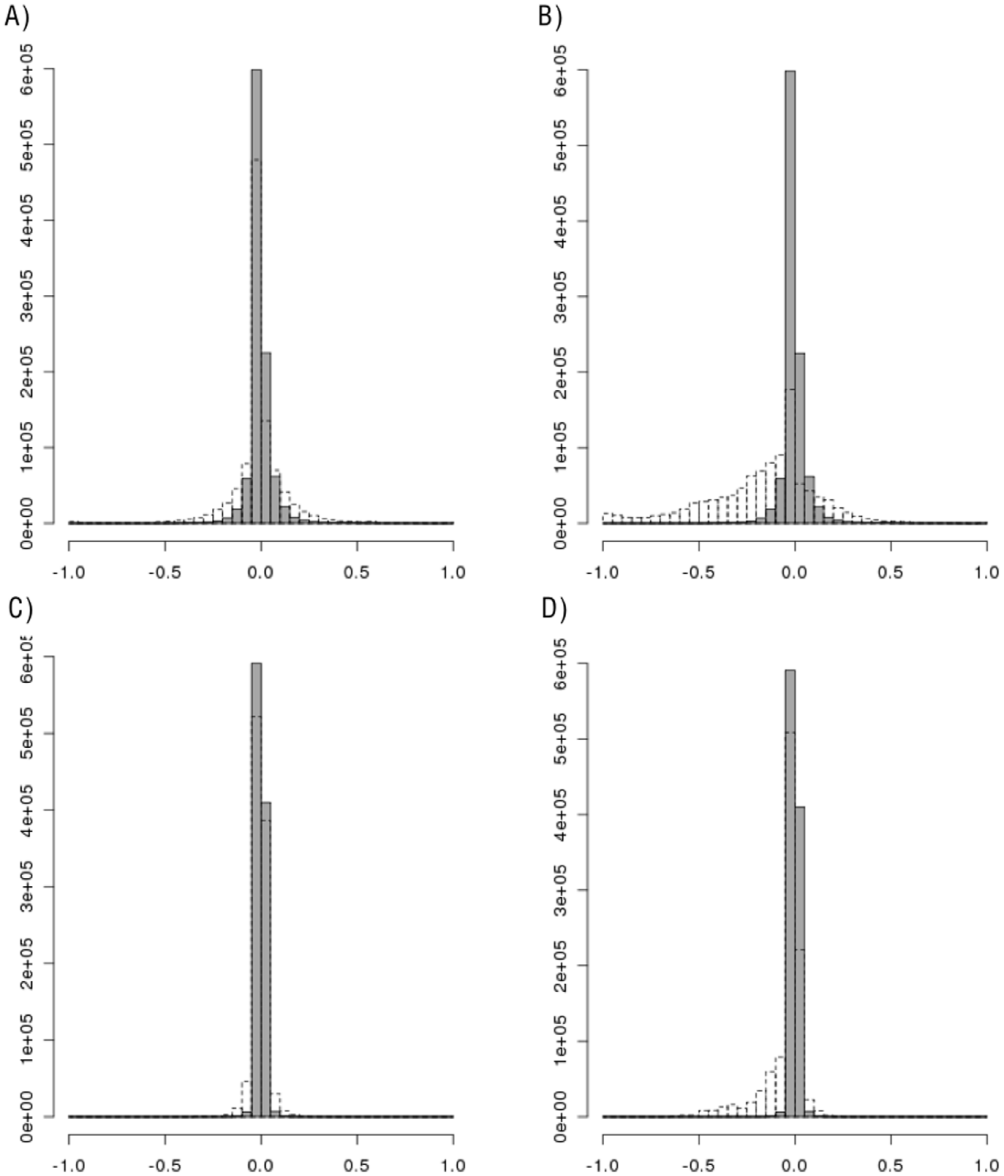
Histograms of the change in LD for each pair of SNPs in Gene Region 1. Histograms of the change in simulated LD from original LD for each pair of SNPs in Gene Region 1 (LD_simulated_ – LD_HapMap_). A) D’, Resampling (gray) vs Hapgen (dotted); B) D’, Resampling (gray) vs HapSim (dotted); C) r^2^, Resampling (gray) vs Hapgen (dotted); D) r^2^, Resampling (gray) vs HapSim (dotted).

## Materials and Methods

### Gene Regions

To ensure generalizability for our comparisons, we used two diverse gene regions. The first gene region is located on chromosome 4 and was defined as 100 Kb from each end of the longest *SNCA* transcript using NCBI36. This definition produced a region of approximately 300 Kb, ranging from 90,765,728 to 91,078,470 bp (Figure S1A). We chose this region because of our research in Parkinson’s Disease (PD). This gene contains rare single nucleotide mutations and duplications within *SNCA* that have been shown to cause PD and, more recently, common variants within *SNCA* that have been shown to be associated with PD [13].

**Figure 3:**
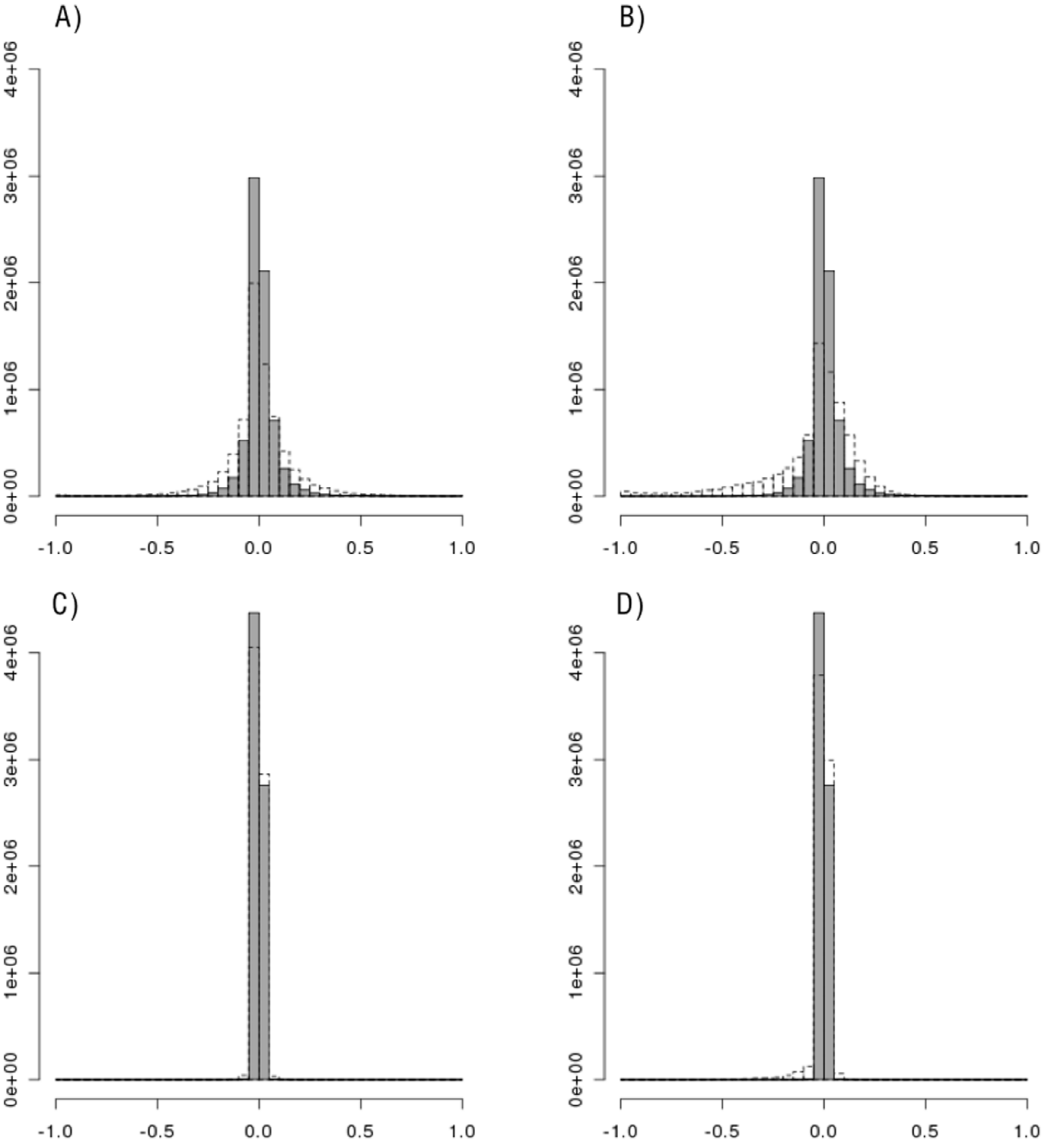
Histograms of the change in LD for each pair of SNPs in Gene Region 2. Histograms of the change in simulated LD from original LD for each pair of SNPs in Gene Region 2 (LD_simulated_ – LD_HapMap_). A) D’, Resampling (gray) vs Hapgen (dotted); B) D’, Resampling (gray) vs HapSim (dotted); C) r^2^, Resampling (gray) vs Hapgen (dotted); D) r^2^, Resampling (gray) vs HapSim (dotted).

**Table 3:** Ratio of replicate SD to original SE for each SNP pair.

		GR1	GR2
		Median (Q1, Q3)	Median (Q1, Q3)
	**Hapgen**	0.7047 (0.5973, 0.8091)	0.6747 (0.0776, 0.7948)
**D’**	**Resampling**	0.3543 (0.2251, 0.4399)	0.3574 (0.2221, 0.4084)
	**HapSim**	0.4249 (0.3168, 0.5427)	0.3506 (0, 0.4165)
	**Hapgen**	0.7886 (0.5264, 1.0360)	0.7164 (0.6215, 0.8524)
**r**	**Resampling**	0.2797 (0.2408, 0.3171)	0.4311 (0.3610, 0.4827)
	**HapSim**	0.3504 (0.2624, 0.6330)	0.4417 (0.3829, 0.4909)

Another region on chromosome 4 has been shown to be associated with Atrial Fibrillation [14], [15]. Also using NCBI36, we defined this second gene region as 500 Kb on either side of the SNP with the lowest p-value from a preliminary Atrial Fibrillation CHARGE+ consortium meta-analysis [16]. This region, also located on chromosome 4, ranges from 111,396,240 to 112,396,240 bp, and contains 445 HapMap Phase III SNPs. The LD plot for each region is displayed in Figure 1.

As described above, we defined the gene regions using two distinct definitions, which produced regions with different lengths, and LD patterns. Some researchers may choose an area around a particular gene, which is similar to how we defined gene region 1, while others may simulate a large section of a chromosome based on some other criterion such as a region surrounding the SNP with the lowest p-value as we do for gene region 2. While the size of the gene regions being simulated and analyzed by other researchers will depend on the definition used to create the region, we believe our gene regions encompass much of the range that would be seen in other studies.

**Figure 4:**
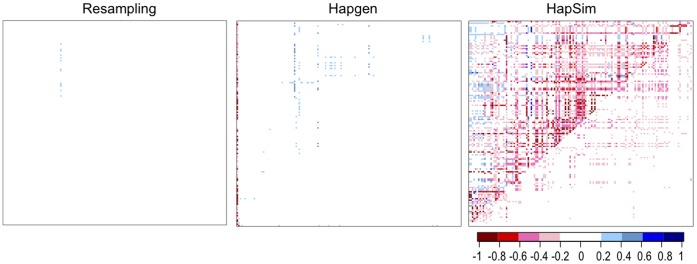
Heat maps of change in median LD for Gene Region 1. Heat maps of change in median simulated LD from original LD in Gene Region 1 (median[LD_simulated_] – LD_HapMap_). Upper left D’, lower right r^2^. Blue indicates a gain in LD; red indicates a loss in LD.

**Figure 5:**
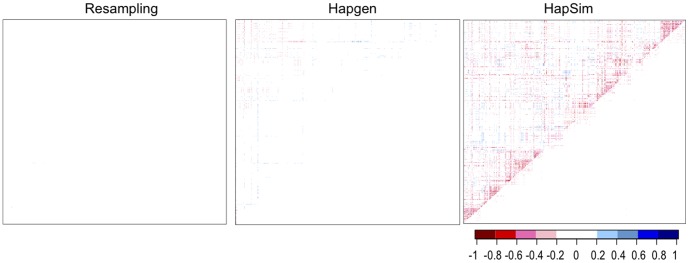
Heat maps of change in median LD for Gene Region 2. Heat maps of change in median simulated LD from original LD in Gene Region 2 (median[LD_simulated_] – LD_HapMap_). Upper left D’, lower right r^2^. Blue indicates a gain in LD; red indicates a loss in LD.

**Table 4:** LD–Gene Region 1*.

	Method	N**	Min	Q1	Median	Q3	Max	Mean	SD
**D’**	Hapgen	1011611	−1.000	−0.030	<0.001	0.021	1.000	−0.004	0.154
	Resampling	1015300	−0.991	−0.006	<0.001	0.010	0.993	0.003	0.058
	HapSim	1015300	−1.000	−0.309	−0.106	0.000	1.000	−0.161	0.288
**r^2^**	Hapgen	1011611	−1.000	−0.008	<0.001	0.006	0.445	−0.002	0.036
	Resampling	1015300	−0.254	−0.003	<0.001	0.003	0.259	<0.001	0.014
	HapSim	1015300	−0.665	−0.049	−0.006	0.000	0.275	−0.046	0.103

* Change in simulated LD from original HapMap sample LD for each pair of SNPs in Gene Region 1 (LD_simulated_ – LD_HapMap_).

** Sum of SNP pairs over all 100 replicates. The number of SNP pairs for Hapgen is not divisible by 100 because monoallelic SNPs were dropped from the LD calculations. Because Hapgen had more variation in MAF, it was more likely that a SNP with a low MAF would become monoallelic in one or more simulation replicates and would thus be dropped from the LD calculations.

**Table 5:** LD–Gene Region 2*.

	Method	N**	Min	Q1	Median	Q3	Max	Mean	SD
**D’**	Hapgen	7002401	−1.000	−0.053	<0.001	0.061	1.000	0.002	0.172
	Resampling	7138597	−0.999	−0.018	<0.001	0.032	1.000	0.009	0.073
	HapSim	7149516	−1.000	−0.102	<0.001	0.070	1.000	−0.050	0.224
**r^2^**	Hapgen	7002401	−0.861	−0.002	<0.001	0.002	0.666	<0.001	0.013
	Resampling	7138597	−0.250	−0.001	<0.001	0.001	0.359	<0.001	0.005
	HapSim	7149516	−0.934	−0.002	<0.001	0.003	0.333	−0.005	0.040

* Change in simulated LD from original HapMap sample LD for each pair of SNPs in Gene Region 2 (LD_simulated_ – LD_HapMap_).

** Sum of SNP pairs over all 100 replicates. The number of SNP pairs is not divisible by 100 because monoallelic SNPs were dropped from the LD calculations. Because Hapgen had more variation in MAF, it was more likely that a SNP with a low MAF would become monoallelic in one or more simulation replicates and thus, more SNP pairs were dropped from the LD calculations than for Resampling or HapSim.

To generate simulated replicates from these gene regions, we used the populations with European ancestry, CEU and TSI, from the HapMap data (Phase III in 2009) [17]. The CEU samples were gathered from Utah in the United States and represent Northern and Western European ancestry. The TSI samples were gathered from Tuscany in Italy. Although the CEU and TSI samples represent two distinct populations, when looked at in the context of other populations world wide, the CEU and TSI samples tend to cluster close to one another apart from Asian or African samples [18]. The HapMap Phase III data consists of 234 & 176 phased haplotypes for the CEU and TSI samples respectively. The gene regions include several lower frequency SNPs in addition to common variants (Information S1).

### Recombination Rates

We used variable recombination rates across each gene region estimated by the HapMap project using McVean et al.’s coalescent method [19].

**Figure 6:**
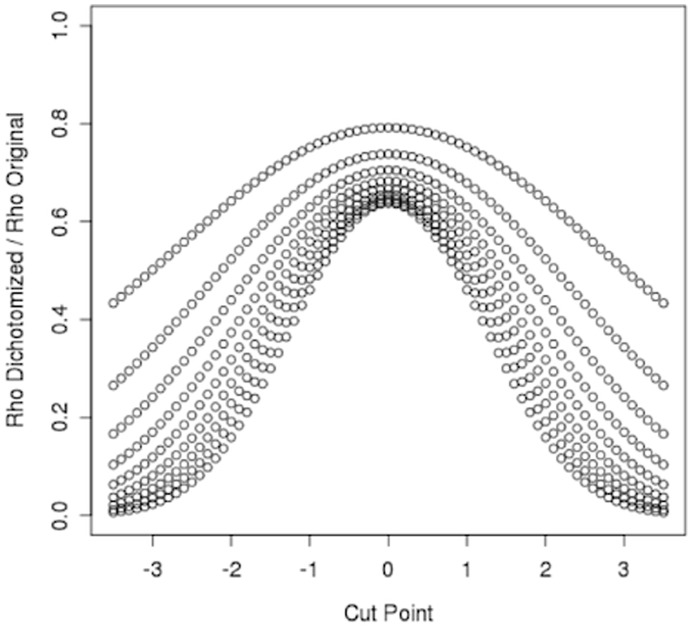
Affect of dichotomizing on the correlation between two normally distributed variables. Correlation between dichotomized variables compared to the original correlation between two normally distributed variables. Each curve represents an original correlation value (*ρ* = 0.1 for the bottom curve to *ρ* = 0.9 for the top curve by 0.1). The same cut point was used for both variables.

### Replicates

For each method and variation, we simulated 100 replicates each consisting of 2,000 subjects.

### Gene Region Simulation Methods

#### Hapgen [6]

We used Hapgen v1.3.0. Hapgen simulates mosaic haplotypes using a Hidden Markov Model to define the probability of continuing on the current haplotype segment or transitioning to a randomly chosen haplotype segment. The transition probabilities are defined by the variable recombination rate across the region as well as the effective population size [20], [21]. Each newly created haplotype is added to the set of original haplotypes from which more haplotypes are created.

#### Extension to Resampling

Given the starting set of haplotypes, we sampled two haplotypes with replacement. We then recombined this pair of haplotypes using a variable recombination rate across the region to specify the probability of recombination occurring at a given chromosomal location. We implemented this method in R [22].

#### HapSim [7]

We used HapSim v0.2. Starting with the original set of haplotype markers, HapSim calculates a covariance matrix assuming a bivariate normal distribution and using the observed joint probability of each haplotype marker pair. HapSim then simulates random vectors from a multivariate normal distribution centered at zero using the previously calculated covariance matrix. Finally, the program transforms the normally distributed vectors back to vectors of binary values using thresholds defined by the observed allele frequency of each marker.

HapSim uses a multivariate normal distribution and the observed MAF and joint probabilities to produce simulated haplotypes. Thus, the parameters used by HapSim are embedded within these choices and are set by using a multivariate normal distribution with a mean of 0, and a covariance matrix estimated using the observed MAF for each marker and joint probabilities for each pair of markers.

It is important to note that the calculated covariance matrix may not be positive definite, which is necessary for the matrix to be used as the covariance matrix for a multivariate normal distribution. When this is the case, HapSim approximates a positive definite version of the covariance matrix by: (1) completing eigenvalue decomposition of the covariance matrix (2) rounding all eigenvalues below a minimum tolerance threshold up to that threshold.

### Comparison of Methods

When comparing methods, we used the following parameters for Hapgen: mutation rate = 0, effective population size = 11,418 (often used for samples of European descent) [6], weight = 1 on the vector of recombination rates, and a randomly chosen starting locus for the Hidden Markov Model for each replicate. We used weight = 1 for the vector of recombination rates for the resampling method as well. HapSim does not easily enable parameter variation. We used both the CEU and TSI samples as the starting haplotype sample for all three methods.

D’ and r^2^ are two common measures of LD. We calculated the D’ and r^2^ using Haploview [23]. (Equations for D’ and r^2^ are shown in Information S1.).

For each replicate, we calculated the LD (D’ or r^2^) for each pair of SNPs. To look at the bias of the LD (D’ or r^2^) we compared the pairwise LD values for each method’s replicates to the original pairwise LD values for the entire region as well as subsets of SNPs in the region by LD, D’ or r^2^, (≤0.2, between 0.2 & 0.8, and ≥0.8) and MAF (≤0.1, between 0.1 & 0.3, and ≥0.3). (The equation for bias is shown in Equation 1.) To visualize this comparison we produced: (1) histograms of the difference between the simulated pairwise LD and original pairwise LD for each pair of SNPs over all replicates and (2) heat map plots of the median change in simulated pairwise LD compared to original pairwise LD. In addition to LD, we compared the distributions of the change between the replicates and the original sample for MAF.
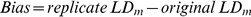
(1)where *LD_m_* is the LD (either D’ or r^2^) for pair of SNPs m

To gain insight into a possible appropriate amount of variation desired between replicates, we calculated the standard error (SE) of the LD estimate from the original data for each SNP pair and compared those with the replicate standard deviation (SD) of the LD estimate for each SNP pair. We used Zapata et al.’s method to estimate the SE for D’ [24]. We calculated and compare the SE and SD of r instead of r^2^ since there exists a commonly used equation to calculate the SE of r (shown in Equation 2) [25]. We calculated the ratio of the replicate SD to the original SE for each SNP pair and compared the methods by looking at the distribution of the ratio across SNP pairs. A ratio value greater than 1 indicates a larger replicate SD compared to the original SE while a ratio value below 1 indicates a lower replicate SD compared to the original SE.
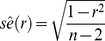
(2)where n is the number of haplotypes

Finally, we calculated the time necessary to simulate 10 replicates using each method for each gene region.

### Parameter Variations

Where possible and using the haplotype sample for Gene Region 1, we varied simulation parameters to study each parameter’s effect on gene region characteristics. When comparing the effects of varying one particular parameter, we kept all other parameters constant at the values used to compare the methods (Hapgen: mutation rate = 0, effective population size = 11,418, recombination rate weight = 1; Extension to resampling: recombination rate weight = 1).

For Hapgen, we compared the mutation rate by varying θ, the modifiable mutation rate parameter to be 0, 1, 2, or 5. Hapgen uses the following formula to model the probability of a mutation occurring at a given SNP where *k* is the total number of original haplotypes and θ is the modifiable mutation rate parameter.
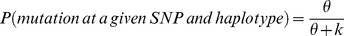
(3)


Using Equation 3 and k = 410 CEU and TSI haplotypes, the resulting probabilities of a mutation at a given SNP for a given haplotype are 0, 0.0024, 0.0049, and 0.0120 for θ = 0, 1, 2, and 5 respectively.

Also for Hapgen, we modified the location of the starting locus of the Hidden Markov Model (random; 90,770,374; 90,955,029; 91,052,395), and the starting haplotype sample (CEU only, TSI only, CEU & TSI). We chose to use the effective population size that is most commonly used for populations of European ancestry (11,418) as well as effective population sizes approximately 1/10^th^ and twice the size as this commonly used value (1,142 and 22,836 respectively) to explore the effects of changing the effective population size parameter in Hapgen.

For Hapgen and the resampling method, we varied the weight by which we multiplied the variable recombination rate vector (0.1, 0.2, 1, 5, 10). The weight changes the recombination rate vector by a multiple of the weight. A weight above one for the recombination rate vector should increase the level of recombination while a weight below one should decrease the level of recombination.

## Results and Discussion

### Comparison of Methods

#### MAF

As shown in Tables 1 and 2, all three methods had similar levels of variation for the change between the simulated and original observed MAF. Most of the time, there was little or no change in each SNP’s MAF, and the change was fairly symmetric as indicated by the quartile values.

#### LD comparisons

As displayed in Figures 2 and 3, Hapgen appears to provide more variation in the change in simulated LD from the original sample LD compared to resampling. All methods had lower replicate SD, on average, for both D’ and r^2^ estimates than the SE estimate from the original data (Table 3). Hapgen produced replicate SD values closest to the original SE estimate. Resampling often produced the lowest ratio of SD to SE estimates indicating that the replicate SD for the LD value for each SNP pair was usually much lower than the estimated SE from the original data.

Even more striking and important, resampling and Hapgen produced little to no bias whereas HapSim appeared to produce a loss in LD across both gene regions as shown in Figures 2, 3, 4, and 5. For Gene Region 1, HapSim had a value for bias below 0 (median D’ = −0.106 & median r^2^ = −0.006, mean D’ = −0.161 & median r^2^ = −0.046) indicating an average loss in LD (Table 4). HapSim’s loss in LD was less extreme for Gene Region 2 (median D’ <0.001 & median r^2^<0.001, mean D’ = −0.050 & median r^2^ = −0.005) where there was a lower starting LD and thus less to lose (Table 5). Both Hapgen and resampling had bias values close to or at 0 indicating little to no bias. HapSim’s loss of LD was seen across all MAFs, but was limited mostly to moderate to high LD pairs (D’ >0.2 or r^2^>0.2) (Figures S6, S7, S8, and S9). This is even more apparent in Gene Region 2 where the loss in LD produced by HapSim was limited exclusively to moderate to high LD pairs. Nonetheless, the bias towards a loss in LD (especially r^2^) for the moderate to high LD groups in Gene Region 2 for HapSim was quite extreme.

Although having to approximate a positive definite version of a matrix when the calculated covariance matrix is not positive definite will introduce error, there is no indication that the error produces a consistent bias. Rather, the error will likely increase the variance. Another, more likely explanation for HapSim’s loss in LD is dichotomizing the vectors of normally distributed variables to vectors of binary values to create the haplotypes. It has been previously shown that dichotomizing normally distributed variables into binary variables decreases the correlation between the variables [26]. To further support this, we calculated the correlation before and after dichotomizing two normally distributed variables. In Figure 6 we show a loss in correlation when dichotomizing using the same threshold for each variable. We continued to see a loss in correlation when different thresholds were used for each variable (Figure S10). Thus, dichotomizing the normally distributed simulated haplotypes is likely the reason the LD decreased for HapSim across the region for the simulated replicates compared to the original sample.

Since association analysis often relies on markers’ correlation with the causal SNP, a loss in the LD across the gene region in the simulation replicates, as seen in HapSim, will decrease the power of most methods to detect association. Further, certain methods may be affected more than others depending on the way the method uses or adjusts for the regional correlation. Thus, the relative order of methods being compared may be affected by this reduction in LD. For example, Principal Component Analysis (PCA) transforms the set of genetic markers into a new set of independent variables (i.e. the principal components). The correlation between the markers will to some degree determine the weight that each marker is given in each principal component. An extreme example would be if all genetic markers were completely independent (i.e. had an LD of zero). The resulting principal components would then each equal one of the genetic markers with a weight of 1 and all of the other genetic markers have a weight of 0. Another method, LASSO regression, controls for the correlation between variables by further shrinking each variable’s regression estimate. As these methods incorporate the regional correlation differently, we would expect that differing LD patterns to in turn have different affects on the resulting power and type-I error of these methods.

For Hapgen, there appeared to be an edge effect for the few SNPs on the left side of Gene Region 1 where there was a large decrease in median D’ values of the simulated replicates compared to the original sample (Figure 4). This large decrease in median D’ was limited to SNP pairs with a large LD (D’ ≥0.8) and a small MAF (MAF ≤0.1) (Figures S6 and S7) indicating that the large change in LD was likely due to highly correlated low frequency SNPs rather than an actual edge effect.

Finally, across all methods and both gene regions, there was a higher degree of variation and bias seen for D’ than for r^2^. This was expected because D’ is more sensitive to low MAF and is estimated to be one in the extreme case where one of the haplotypes has an estimated frequency of zero.

#### Run Time

As shown in Information S1, Hapgen was more than 10x faster than the other simulation methods producing 10 replicates in less than 10 seconds for each gene region. For simulation designs that require tens or hundreds of thousands of replicates, Hapgen would likely require hours while the other methods would likely require days.

### Parameter Variations

#### Starting haplotype sample (Information S1)

Changing the starting sample of haplotypes from both CEU and TSI samples to either CEU only or TSI only samples had very little effect on the variance or bias of the LD distributions although the two smaller samples (CEU only and TSI only) appeared to have slightly more variation compared to the larger sample (both CEU and TSI). This is expected, since every haplotype section is more likely to be drawn from the smaller sample of haplotypes and, thus, the replicate sample more often contains identical haplotype sections, which prevents much decay of LD.

The results seen here were not very sensitive to starting with a different sample of haplotypes. Nonetheless, we recommend using as large a starting sample of haplotypes as possible as long as the samples are representative of the desired population.

#### Mutation rate variation (Information S1)

Increasing the mutation rate led to a loss in LD for the simulated replicates compared to the original sample (Figure S2). This is expected as increasing the mutation rate increases the likelihood that an existing haplotype is changed, thus decreasing the LD between markers.

We recommend taking into consideration the sample size when choosing Hapgen’s mutation rate parameter, θ. θ is equal to the expected number of mutations at each SNP for the sample of haplotypes. As we may expect a larger number of mutations in a larger sample of haplotypes, we may want to increase θ accordingly. In addition, if we have a particular interest in rare variants, we may also want to increase θ to introduce more rare variants.

#### Effective population size variation (Information S1)

Hapgen developers recommend using 11418, 17469, and 14269 for samples of European (HapMap CEPH), African (HapMap Yoruban), and Asian (HapMap Japanese and Chinese) descent respectively [6]. Depending on the time period and population used for estimation, effective population size estimates in the literature range from below 2,000 to about 21,000 [27], [28], [29]. The estimates that we use to compare the effects of changing the effective population size (1142, 11418, and 22836) cover this wide range of effective population size estimates.

The effective population size is the number of mating individuals in a population that will produce the same allele frequency distribution as that observed in the entire population assuming that all individuals in the effective population mate at random and have an equal chance of passing along their genetic information [20], [21]. As the effective population size increases so should the gene region haplotype variation thus decreasing the gene region LD. Here we found that changing the effective population size by a factor of 10 had a small inverse effect on LD: decreasing the effective population size produced higher simulated LD while increasing the effective population size produced lower simulated LD (Figure S3). This is expected, as the formula implemented by Hapgen inversely relates the effective population size to the transition probability in the Hidden Markov Model.

#### Starting locus variation (Information S1)

Using a different locus as the starting point for the Hidden Markov Model in Hapgen did not produce any notable change in variation or bias of the replicates. We recommend using a randomly chosen starting location, as the starting locus should not make any difference when only control or general population haplotypes are simulated.

#### Recombination rate variation (Information S1)

Changing the recombination rate had a visible effect on the difference in LD between the replicates and the original sample (Figures S4 and S5). Increasing the recombination rate increased how often an existing haplotype was altered, thus decreasing the LD between markers. Given a high enough recombination rate, all LD within a region would be lost. Interestingly, as we decreased the recombination rate, we saw a slight shift towards a gain in LD for the replicates compared to the original sample. The reason for the slight gain in LD is easiest explained through an example of a recombination rate of zero. When the recombination rate is zero, or so low that it is essential zero, haplotypes are chosen with replacement from the original sample to create the sample for each replicate. Often, especially when the sample size of the replicates is larger than the original sample size, the same haplotypes appear several times in a replicate thus increasing the LD [32]. The decrease in LD seen by increasing the variable recombination rate was seen in both Hapgen and resampling, although it was much greater for Hapgen.

Unless the user specifically intends to alter the LD within a gene region, we recommend using the recombination rates estimated by the Hap Map project using McVean et al.’s method [19] with out any weight (i.e. weight = 1) on the vector of recombination rates.

### Generalizability of Gene Regions

As previously stated, since the methods examined use real data they are likely to give a representative picture of the complex underlying LD structure in a gene region. However, it is important to note the sample will only include variation from the particular gene region and population from which the starting sample was gathered. Nonetheless, simulating from a gene region of interest is likely to at least be representative of the particular gene region and is less dependent on simulation parameters used in alternative genetic simulation methods of backwards and forward time.

### Applicability to Sequence Data

Recently, many research groups have started to use sequence data to search for genetic associations for variants with low or rare MAFs [30], [31]. We believe these methods are applicable to sequence data. However, these and other methods that simulate from a starting sample of sequenced haplotypes may over represent rare markers present in the original haplotype sample especially when the simulated sample is much larger than the original haplotype sample [32]. Additionally, these methods may under represent rare markers not present in the original sample of haplotypes. We believe these methods may over or under represent rare markers because the starting sample of haplotypes is not completely representative of the entire population of haplotypes, especially for rare markers. Using the mutation rate parameter in Hapgen may help to alleviate the latter issue by adding in new rare variants to the simulation samples not seen in the original haplotypes. More research is warranted.

### Conclusions

We have implemented in parallel three methods for simulating a gene region from a sample of existing haplotypes. We compared these methods using two gene regions that differed in size, LD strength and pattern, and distribution of MAF. Thus, we believe our results and conclusions are applicable to most other gene regions across the genome.

Our goal was to find an adequate simulation method by comparing the LD measures (D’ and r^2^), and MAF for each of the methods. Producing gene region simulations with a representative LD structure is essential for appropriately comparing genetic association analysis methods, which rely on the LD in the region to find risk signals. Based on our findings, we do not recommend using HapSim as the simulation program produces samples of haplotypes with lower LD, on average, compared to the original haplotype set, especially for gene regions with moderate to high LD. Further, since HapSim does not incorporate parameters, it is both less subjective as well as less modifiable. This is an important consideration when simulating gene regions with rare variants where we may want to introduce additional rare variants by using a mutation rate parameter.

Although our simple resampling method does not introduce bias, the variation introduced across the replicates is quite small and may not provide enough sampling variability between replicates to make a generalizable simulation study. The variability of the resampling method could possibly be increased with further modifications such as completing the resampling process over multiple generations.

Among the gene region simulation methods reviewed here, we recommend using Hapgen. Hapgen provides ample variation between replicates while retaining the LD structure of the gene region and does not introduce an overall loss or gain in LD. In addition, Hapgen is easy to use and provides options for changing additional parameters such as a recombination rate or mutation rate, enabling users to modify the simulation settings to better model a particular population or level of variation in the haplotypes.

## Supporting Information

Figure S1 **SNAP P-value Plots of Gene Region Motivating Examples.**(A) Gene Region 1: SNCA region defined as 100 Kb outside of the longest transcript using data from Pankratz et al. [13] and (B) Gene Region 2: chromosome 4 AF peak defined as 500 Kb from the SNP with the lowest p-value using preliminary CHARGE + consortium data.
(TIFF)

Figure S2 **Mutation Rate (MR) variation.** Histograms of the change in simulated LD from original LD for each pair of SNPs in Gene Region 1 using Hapgen (LD_simulated_ – LD_HapMap_). A) D’, MR = 0 (gray) vs MR = 1 (dotted); B) D’, MR = 0 (gray) vs MR = 5 (dotted); C) r^2^, MR = 0 (gray) vs MR = 1 (dotted); D) r^2^, MR = 0 (gray) vs MR = 5 (dotted).
(TIFF)

Figure S3 **Effective Population Size (EPS) variation.** Histograms of the change in simulated LD from original LD for each pair of SNPs in Gene Region 1 using Hapgen (LD_simulated_ – LD_HapMap_). A) D’, EPS = 11,418 (gray) vs EPS = 1,142 (dotted); B) D’, EPS = 11,418 (gray) vs EPS = 22,836 (dotted); C) r^2^, EPS = 11,418 (gray) vs EPS = 1,142 (dotted); D) r^2^, EPS = 11,418 (gray) vs EPS = 22,836 (dotted).
(TIFF)

Figure S4 **Recombination Rate Weight variation: Hapgen.** Histograms of the change in simulated LD from original LD for each pair of SNPs in Gene Region 1 using Hapgen (LD_simulated_ – LD_HapMap_). A) D’, RRW = 1 (gray) vs RRW = 0.1 (dotted); B) D’, RRW = 1 (gray) vs RRW = 10 (dotted); C) r^2^, RRW = 1 (gray) vs RRW = 0.1 (dotted); D) r^2^, RRW = 1 (gray) vs RRW = 10 (dotted).
(TIFF)

Figure S5 **Recombination Rate Weight variation: Resampling.** Histograms of the change in simulated LD from original LD for each pair of SNPs in Gene Region 1 using Resampling (LD_simulated_ – LD_HapMap_). A) D’, RRW = 1 (gray) vs RRW = 0.1 (dotted); B) D’, RRW = 1 (gray) vs RRW = 10 (dotted); C) r^2^, RRW = 1 (gray) vs RRW = 0.1 (dotted); D) r^2^, RRW = 1 (gray) vs RRW = 10 (dotted).
(TIFF)

Figure S6 **Heat maps of change in median LD for Gene Region 1 by LD Group.** Heat maps of change in median simulated LD from original LD in Gene Region 1 by LD group (median[LD_simulated_] – LD_HapMap_). Upper left D’, lower right r^2^. Blue indicates a gain in LD; red indicates a loss in LD.
(TIFF)

Figure S7 **Heat maps of change in median LD for Gene Region 1 by MAF Group.** Heat maps of change in median simulated LD from original LD in Gene Region 1 by MAF group (median[LD_simulated_] – LD_HapMap_). Markers are only included in each plot if both markers fall in the MAF group. Upper left D’, lower right r^2^. Blue indicates a gain in LD; red indicates a loss in LD.
(TIFF)

Figure S8 **Heat maps of change in median LD for Gene Region 2 by LD Group.** Heat maps of change in median simulated LD from original LD in Gene Region 2 by LD group (median[LD_simulated_] – LD_HapMap_). Upper left D’, lower right r^2^. Blue indicates a gain in LD; red indicates a loss in LD.
(TIFF)

Figure S9 **Heat maps of change in median LD for Gene Region 2 by MAF Group.** Heat maps of change in median simulated LD from original LD in Gene Region 2 by MAF group (median[LD_simulated_] – LD_HapMap_). Markers are only included in each plot if both markers fall in the MAF group. Upper left D’, lower right r^2^. Blue indicates a gain in LD; red indicates a loss in LD.
(TIFF)

Figure S10 **Affect of dichotomizing on the correlation between two normally distributed variables with different cut points.** Dichotomized correlation compared to original correlation. Each curve represents an original correlation value (ρ = 0.1 for the lowest peaked curve to ρ = 0.9 for the highest peaked curve by 0.1). One cut point varied along the x-axis while the other was held constant for each plot. A) c_2_ = −3, B) c_2_ = −1.5, C) c_2_ = 0, D) c_2_ = 1.5, E) c_2_ = 3
(TIFF)

Information S1 **Includes LD equations and supplemental tables.**
(DOC)
