# Supplementary figures and images for: A Comparison of Gene Region Simulation Methods
Source: PLoS One. 2012 Jul 18;7(7):e40925. doi: 10.1371/journal.pone.0040925 (PMC3399793; doi:10.1371/journal.pone.0040925)

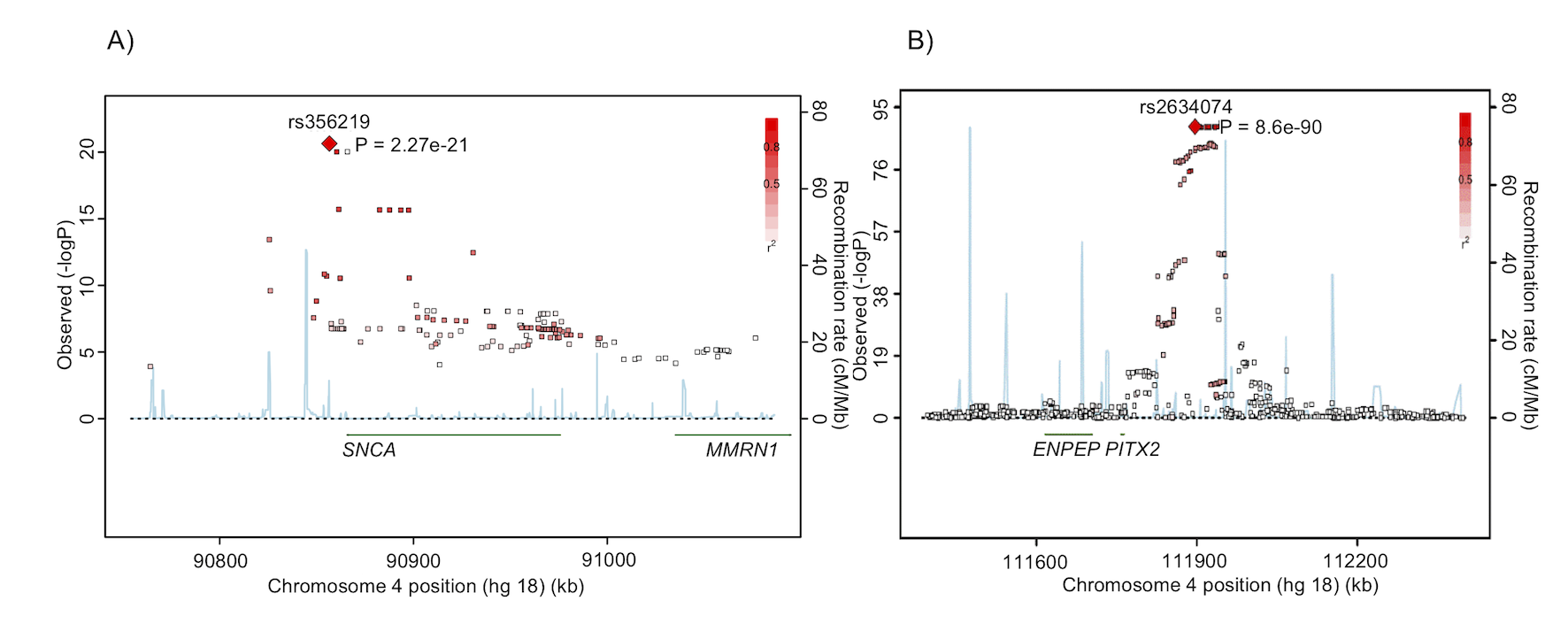

Supplement: Figure S1 — SNAP P-value Plots of Gene Region Motivating Examples.(A) Gene Region 1: SNCA region defined as 100 Kb outside of the longest transcript using data from Pankratz et al. [13] and (B) Gene Region 2: chromosome 4 AF peak defined as 500 Kb from the SNP with the lowest p-value using preliminary CHARGE + consortium data. (TIFF) [file pone.0040925.s001.tiff]

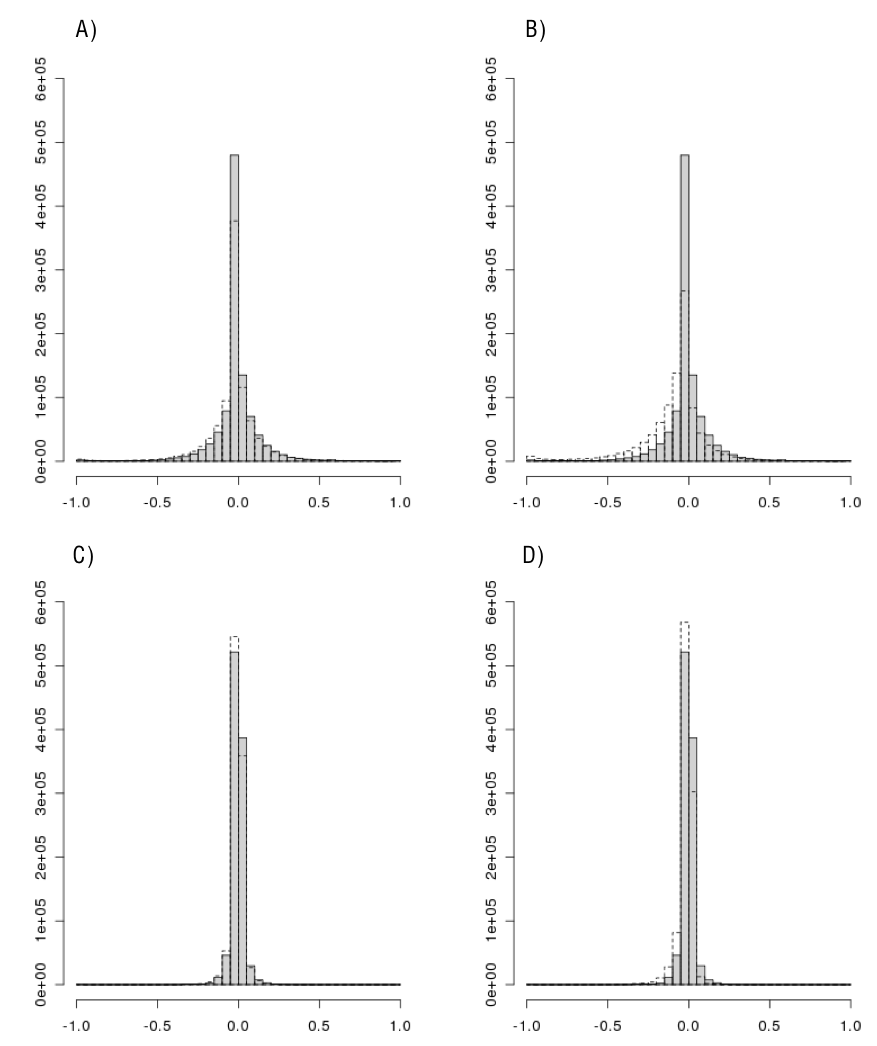

Supplement: Figure S2 — Mutation Rate (MR) variation. Histograms of the change in simulated LD from original LD for each pair of SNPs in Gene Region 1 using Hapgen (LDsimulated – LDHapMap). A) D’, MR = 0 (gray) vs MR = 1 (dotted); B) D’, MR = 0 (gray) vs MR = 5 (dotted); C) r2, MR = 0 (gray) vs MR = 1 (dotted); D) r2, MR = 0 (gray) vs MR = 5 (dotted). (TIFF) [file pone.0040925.s002.tiff]

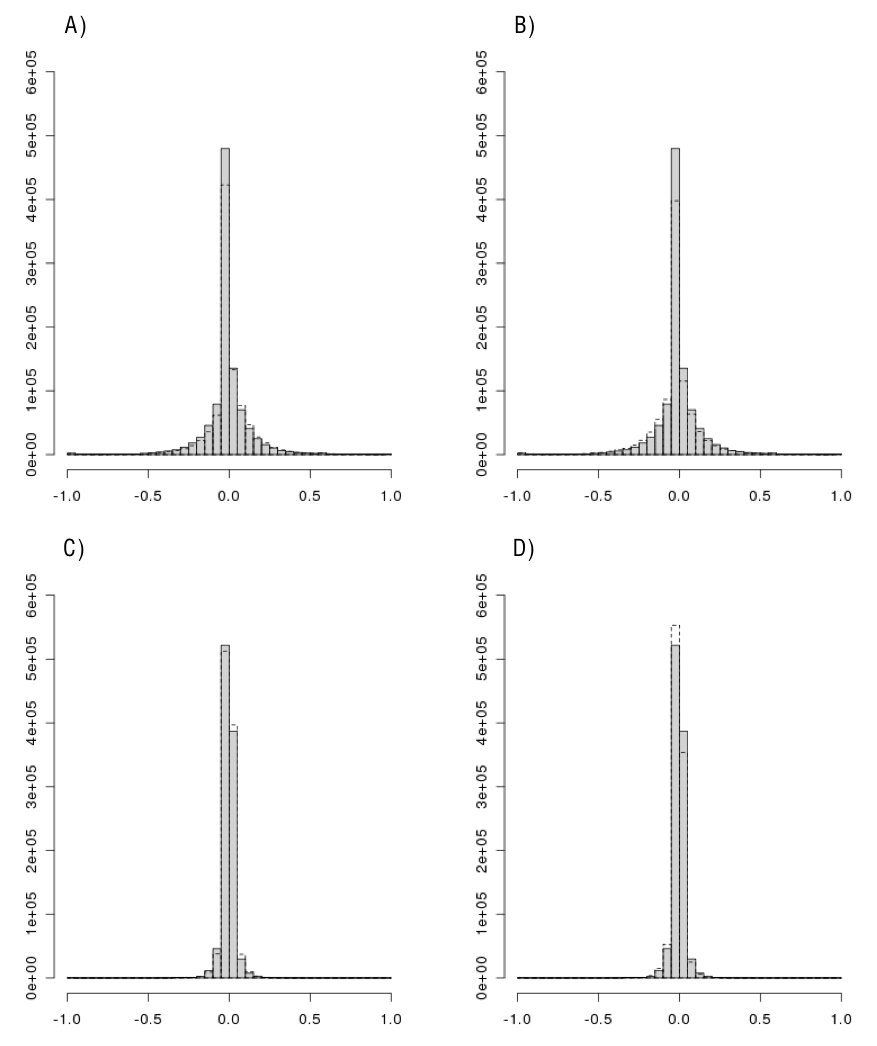

Supplement: Figure S3 — Effective Population Size (EPS) variation. Histograms of the change in simulated LD from original LD for each pair of SNPs in Gene Region 1 using Hapgen (LDsimulated – LDHapMap). A) D’, EPS = 11,418 (gray) vs EPS = 1,142 (dotted); B) D’, EPS = 11,418 (gray) vs EPS = 22,836 (dotted); C) r2, EPS = 11,418 (gray) vs EPS = 1,142 (dotted); D) r2, EPS = 11,418 (gray) vs EPS = 22,836 (dotted). (TIFF) [file pone.0040925.s003.tiff]

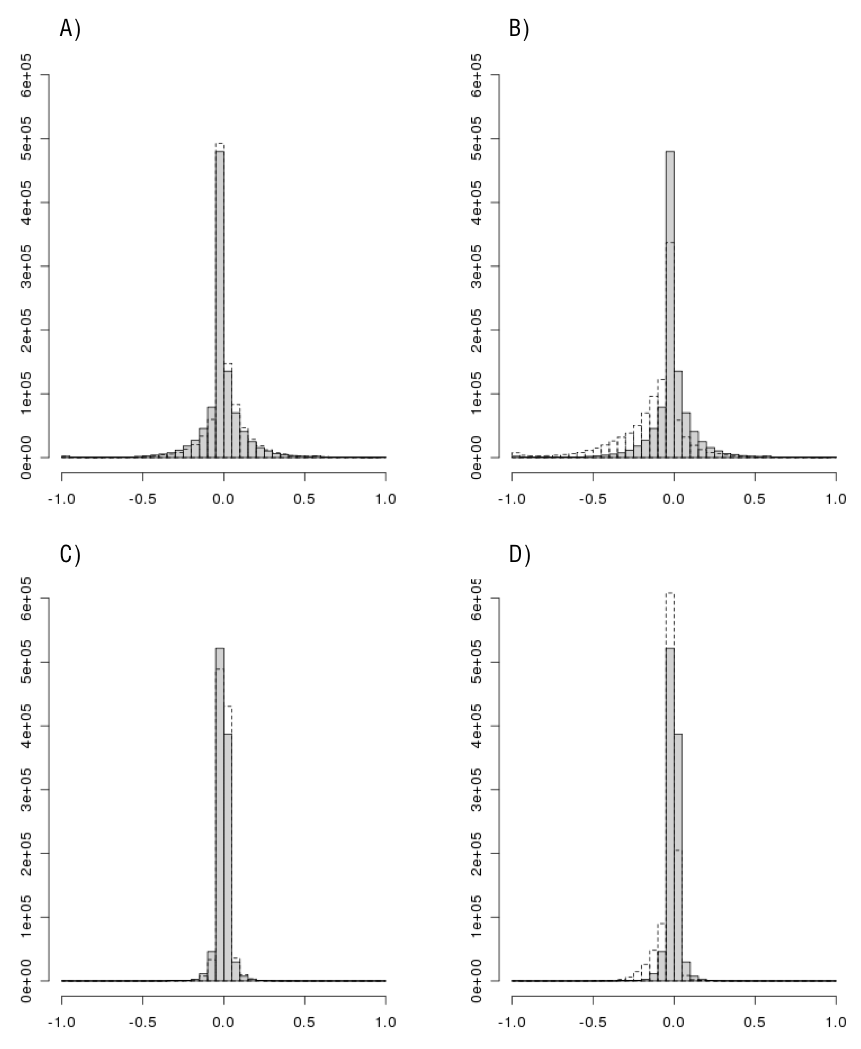

Supplement: Figure S4 — Recombination Rate Weight variation: Hapgen. Histograms of the change in simulated LD from original LD for each pair of SNPs in Gene Region 1 using Hapgen (LDsimulated – LDHapMap). A) D’, RRW = 1 (gray) vs RRW = 0.1 (dotted); B) D’, RRW = 1 (gray) vs RRW = 10 (dotted); C) r2, RRW = 1 (gray) vs RRW = 0.1 (dotted); D) r2, RRW = 1 (gray) vs RRW = 10 (dotted). (TIFF) [file pone.0040925.s004.tiff]

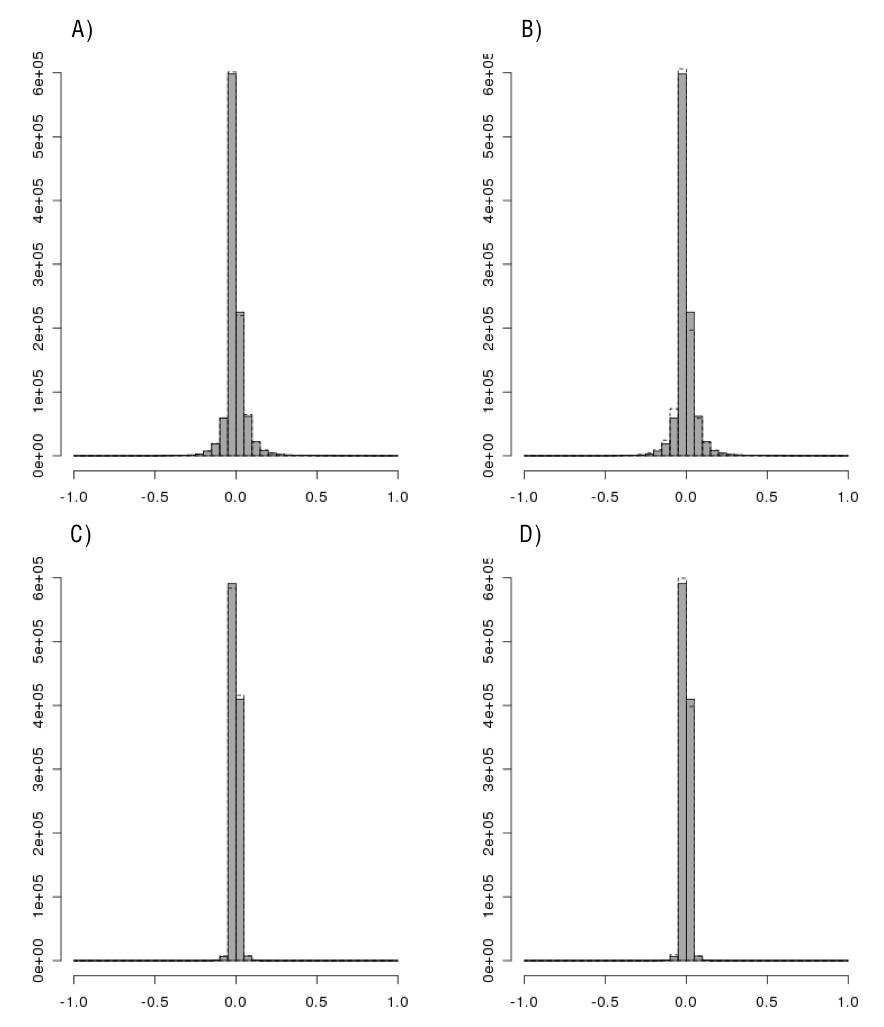

Supplement: Figure S5 — Recombination Rate Weight variation: Resampling. Histograms of the change in simulated LD from original LD for each pair of SNPs in Gene Region 1 using Resampling (LDsimulated – LDHapMap). A) D’, RRW = 1 (gray) vs RRW = 0.1 (dotted); B) D’, RRW = 1 (gray) vs RRW = 10 (dotted); C) r2, RRW = 1 (gray) vs RRW = 0.1 (dotted); D) r2, RRW = 1 (gray) vs RRW = 10 (dotted). (TIFF) [file pone.0040925.s005.tiff]

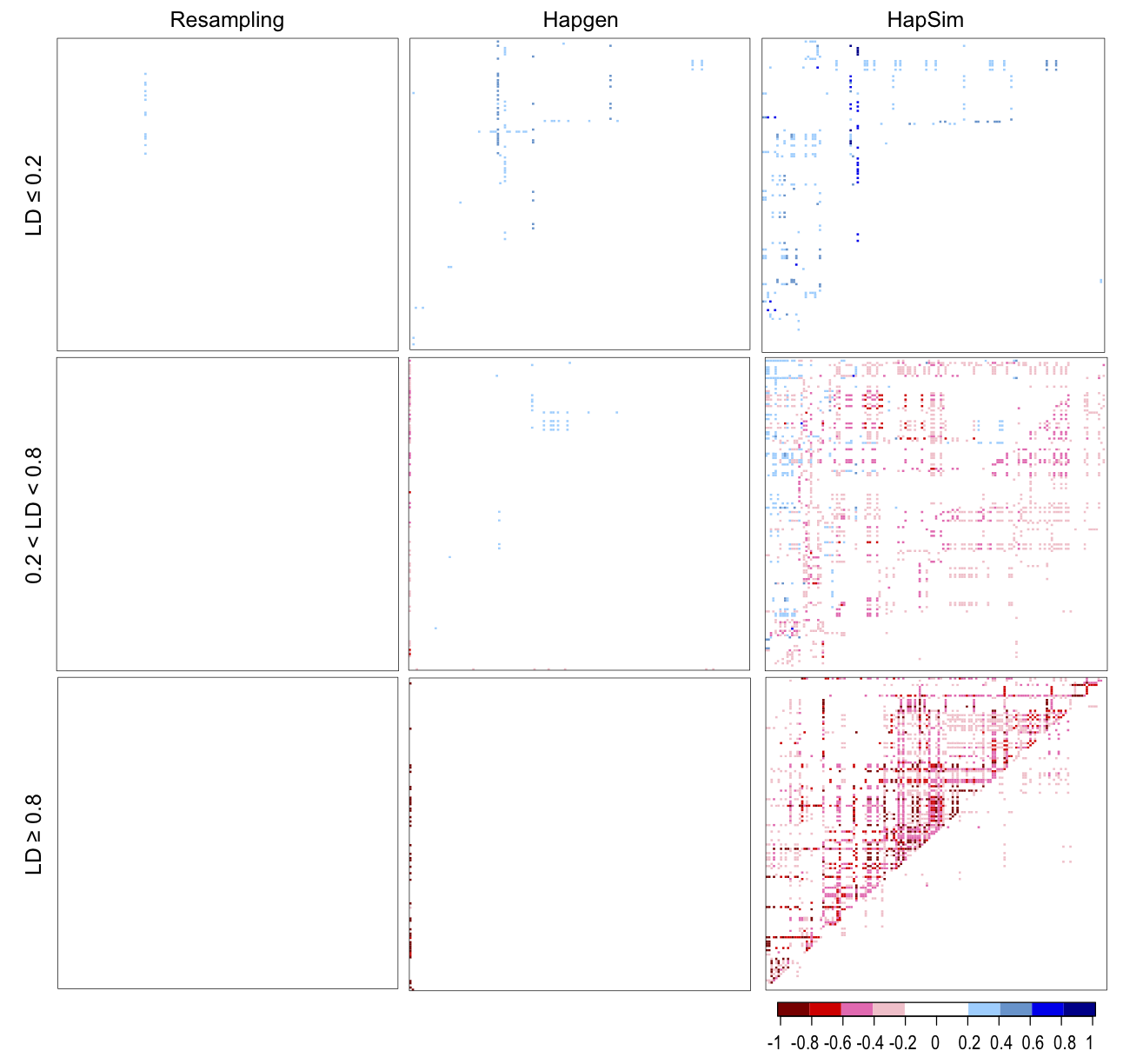

Supplement: Figure S6 — Heat maps of change in median LD for Gene Region 1 by LD Group. Heat maps of change in median simulated LD from original LD in Gene Region 1 by LD group (median[LDsimulated] – LDHapMap). Upper left D’, lower right r2. Blue indicates a gain in LD; red indicates a loss in LD. (TIFF) [file pone.0040925.s006.tiff]

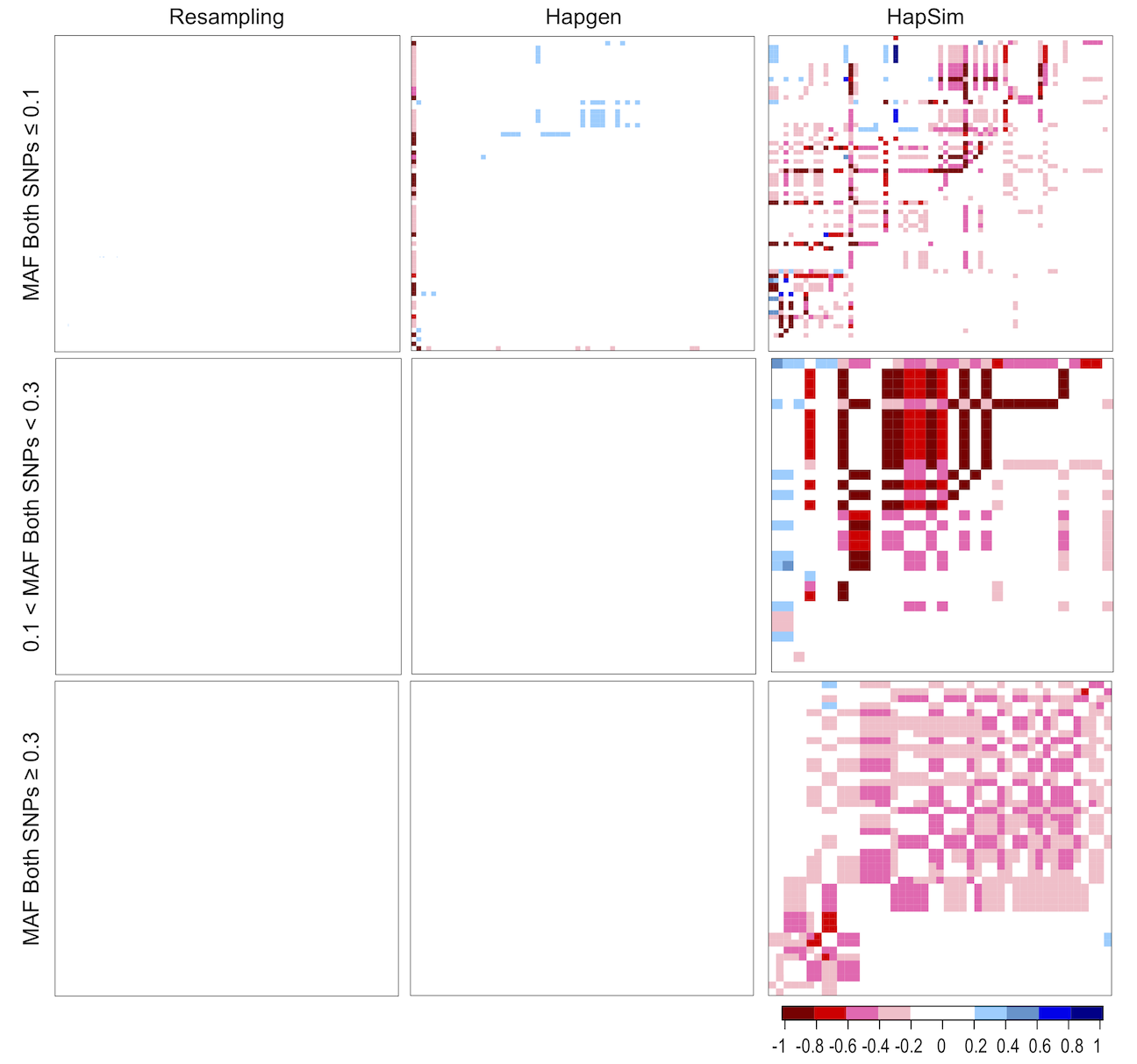

Supplement: Figure S7 — Heat maps of change in median LD for Gene Region 1 by MAF Group. Heat maps of change in median simulated LD from original LD in Gene Region 1 by MAF group (median[LDsimulated] – LDHapMap). Markers are only included in each plot if both markers fall in the MAF group. Upper left D’, lower right r2. Blue indicates a gain in LD; red indicates a loss in LD. (TIFF) [file pone.0040925.s007.tiff]

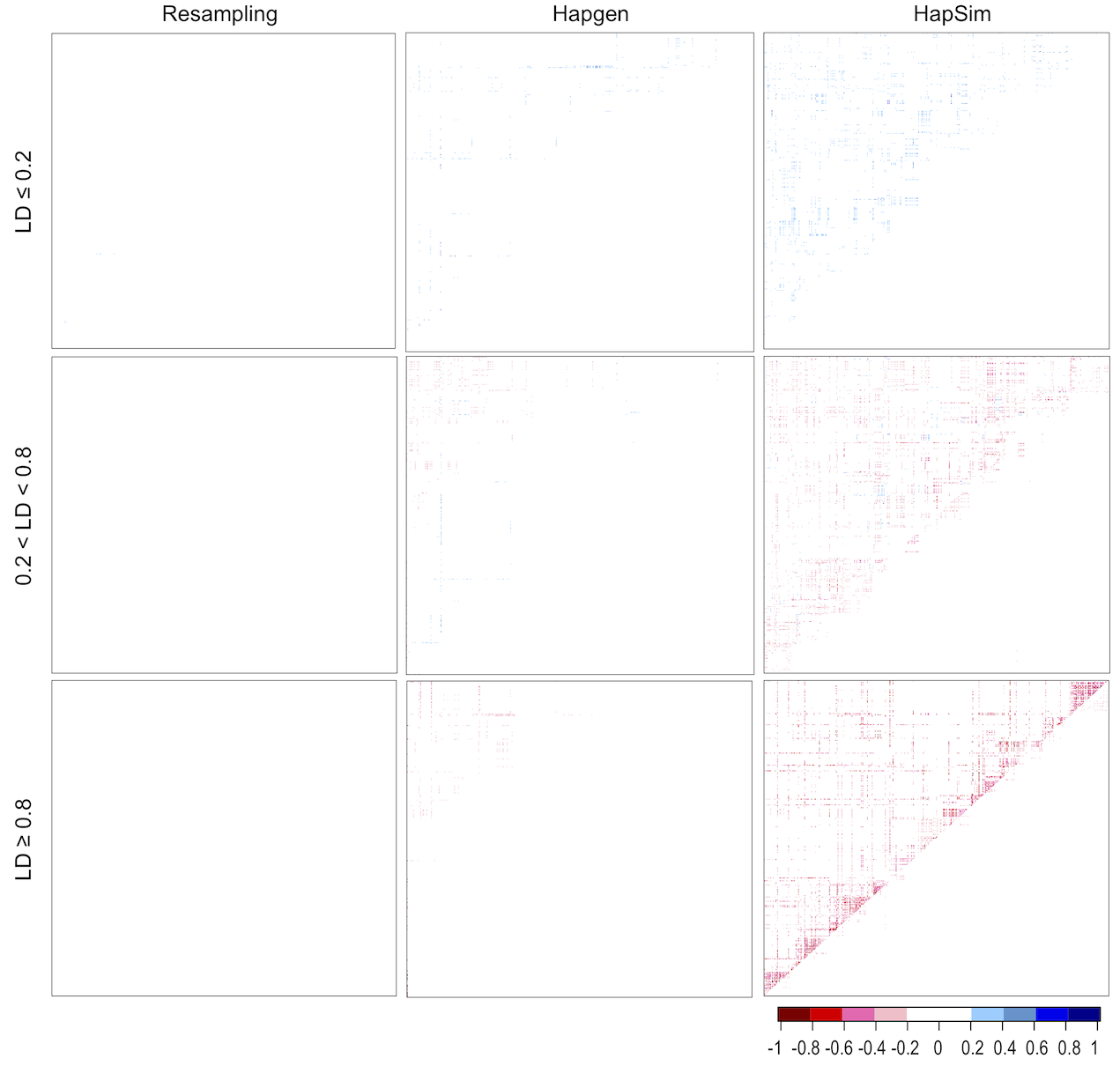

Supplement: Figure S8 — Heat maps of change in median LD for Gene Region 2 by LD Group. Heat maps of change in median simulated LD from original LD in Gene Region 2 by LD group (median[LDsimulated] – LDHapMap). Upper left D’, lower right r2. Blue indicates a gain in LD; red indicates a loss in LD. (TIFF) [file pone.0040925.s008.tiff]

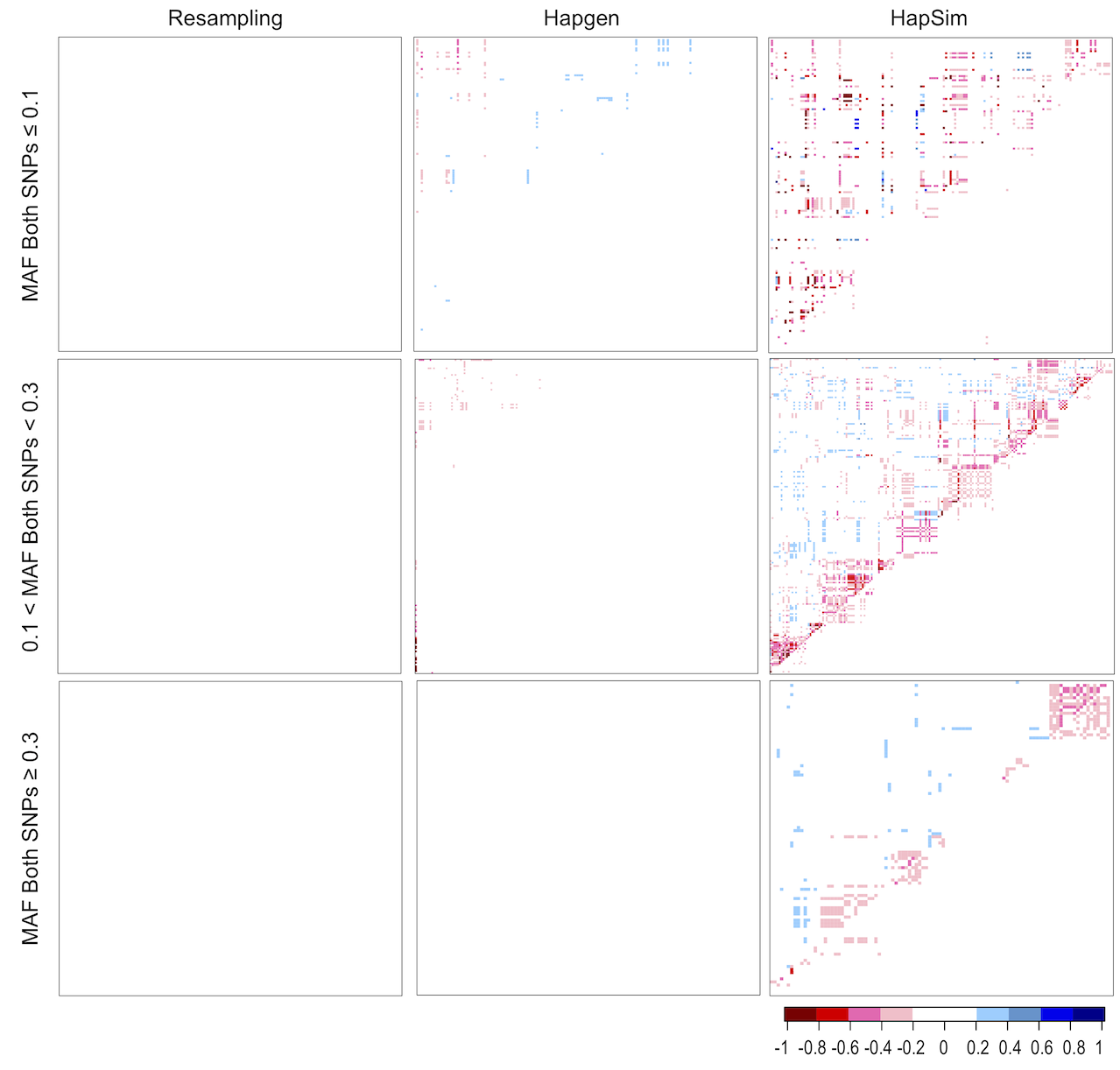

Supplement: Figure S9 — Heat maps of change in median LD for Gene Region 2 by MAF Group. Heat maps of change in median simulated LD from original LD in Gene Region 2 by MAF group (median[LDsimulated] – LDHapMap). Markers are only included in each plot if both markers fall in the MAF group. Upper left D’, lower right r2. Blue indicates a gain in LD; red indicates a loss in LD. (TIFF) [file pone.0040925.s009.tiff]

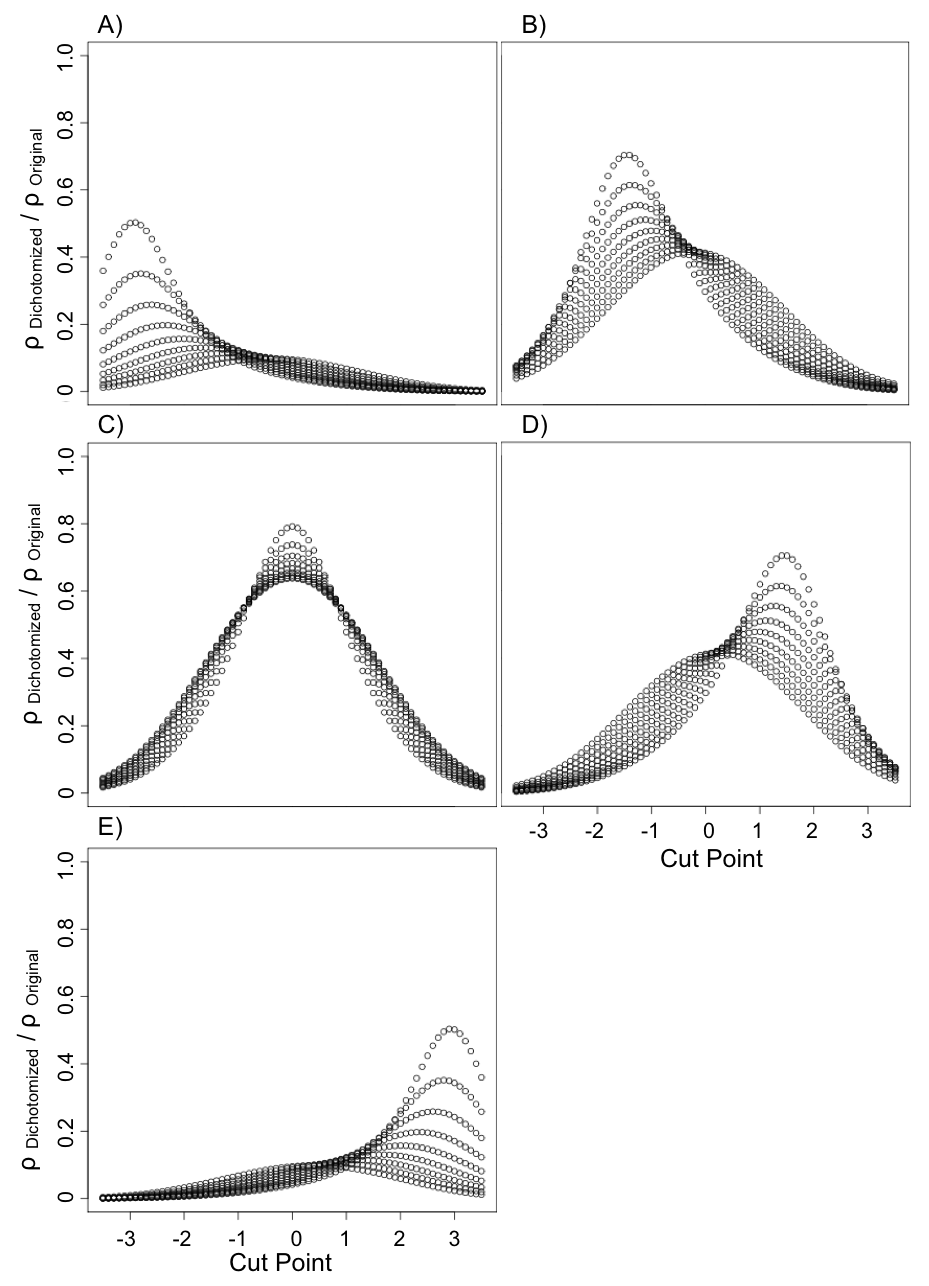

Supplement: Figure S10 — Affect of dichotomizing on the correlation between two normally distributed variables with different cut points. Dichotomized correlation compared to original correlation. Each curve represents an original correlation value (ρ = 0.1 for the lowest peaked curve to ρ = 0.9 for the highest peaked curve by 0.1). One cut point varied along the x-axis while the other was held constant for each plot. A) c2 = −3, B) c2 = −1.5, C) c2 = 0, D) c2 = 1.5, E) c2 = 3 (TIFF) [file pone.0040925.s010.tiff]
