# Supplementary material for: A Comparison of Gene Region Simulation Methods
Source: PLoS One. 2012 Jul 18;7(7):e40925. doi: 10.1371/journal.pone.0040925 (PMC3399793; doi:10.1371/journal.pone.0040925)
Supplement: Information S1 — Includes LD equations and supplemental tables. (DOC) [file pone.0040925.s011.doc]

**Supplemental LD Equations**

D’ and r^2^ can be calculated using the following equations given two markers, A and B, with alleles A, a and B, b as the major and minor alleles for markers A and B respectively.

$$D'=\frac{D}{D_{\max}}$$

$$D=P_{AB}-P_{A}\cdot P_{B}$$

$$D_{\max}=\left\{\begin{matrix} \min\left(P_{A}P_{B},P_{a}P_{b}\right), when D>0 \\ \min\left(P_{A}P_{b},P_{a}P_{B}\right), when D<0 \end{matrix}\right.$$

$$r^{2}=\frac{D^{2}}{P_{A}\cdot P_{B}\cdot P_{a}\cdot P_{b}}$$

**Supplemental Tables**

**Table S1.** MAF Distribution of Gene Region SNPs.

| **Gene**  **Region** | **Monoallelic**  **N (prop.)** | **0 < MAF < 0.01**  **N (prop.)** | **0.01 ≤ MAF ≤ 0.05 N (prop.)** | **MAF > 0.05**  **N (prop.)** | **Total**  **N** |
| --- | --- | --- | --- | --- | --- |
| 1 | 2 (0.0138) | 4 (0.0276) | 33 (0.2276) | 106 (0.7310) | 145 |
| 2 | 66 (0.1483) | 19 (0.0427) | 23 (0.0517) | 337 (0.7573) | 445 |

Table S2. Run Time Comparison. Time to complete 10 replicates.

|  | Time | |
| --- | --- | --- |
|  | Gene Region 1 | Gene Region 2 |
| Hapgen | 3 sec. | 8 sec. |
| HapSim | 39 sec. | 1 min. 53 sec. |
| With Replacement | 57 sec. | 1 min. 43 sec. |

* The programs were run on a Dell blade server with 2 x Quad Core Intel(R)

Xeon(R) CPUs E5345 @ 2.33GHz and 32 GB of RAM running CentOS release 5.3

Linux Distribution.

**Table S3. Starting SampleVariation—Gene Region 1***

|  | Starting Sample | N** | Min | Q1 | Median | Q3 | Max | Mean | SD |
| --- | --- | --- | --- | --- | --- | --- | --- | --- | --- |
|  | CEU & TSI | 930525 | -1.000 | -0.037 | < 0.001 | 0.028 | 1.000 | -0.004 | 0.154 |
| D’ | CEU Only | 690300 | -1.000 | -0.085 | < 0.001 | 0.014 | 1.000 | -0.038 | 0.215 |
|  | TSI Only | 919692 | -1.000 | -0.032 | < 0.001 | 0.081 | 1.000 | 0.025 | 0.224 |
|  | CEU & TSI | 1011611 | -1.000 | -0.008 | < 0.001 | 0.006 | 0.445 | -0.002 | 0.036 |
| r^2^ | CEU Only | 690300 | -1.000 | -0.020 | -0.002 | 0.008 | 0.567 | -0.008 | 0.060 |
|  | TSI Only | 996692 | -1.000 | -0.011 | < 0.001 | 0.012 | 0.857 | < 0.001 | 0.057 |

*Change in simulated LD from original HapMap sample LD for each pair of SNPs in Gene Region 1 (LD_simulated_ – LD_HapMap_).

**Sum of SNP pairs over all 100 replicates. The number of SNP pairs is not divisible by 100 because monoallelic SNPs were dropped from the LD calculations.

**Table S4. Mutation Rate Variation—Gene Region 1***

|  | Mutation Rate | N** | Min | Q1 | Median | Q3 | Max | Mean | SD |
| --- | --- | --- | --- | --- | --- | --- | --- | --- | --- |
| D’ | 0 | 930525 | -1.000 | -0.037 | < 0.001 | 0.028 | 1.000 | -0.004 | 0.154 |
|  | 1 | 932468 | -1.000 | -0.065 | < 0.001 | 0.019 | 1.000 | -0.024 | 0.171 |
|  | 2 | 933374 | -1.000 | -0.091 | -0.012 | 0.012 | 1.000 | -0.043 | 0.188 |
|  | 5 | 933800 | -1.000 | -0.150 | -0.048 | 0.000 | 1.000 | -0.091 | 0.213 |
| r^2^ | 0 | 1011611 | -1.000 | -0.008 | < 0.001 | 0.006 | 0.445 | -0.002 | 0.036 |
|  | 1 | 1013738 | -1.000 | -0.011 | -0.001 | 0.005 | 0.481 | -0.005 | 0.037 |
|  | 2 | 1014874 | -1.000 | -0.013 | -0.001 | 0.004 | 0.397 | -0.008 | 0.039 |
|  | 5 | 1015300 | -1.000 | -0.019 | -0.003 | 0.002 | 0.443 | -0.016 | 0.046 |

*Change in simulated LD from original HapMap sample LD for each pair of SNPs in Gene Region 1

(LD_simulated_ – LD_HapMap_).

**Sum of SNP pairs over all 100 replicates. The number of SNP pairs is not divisible by 100 because monoallelic SNPs were dropped from the LD calculations.

Table S5. Effective Population Size Variation—Gene Region 1*

|  | Effective Population Size | N** | Min | Q1 | Median | Q3 | Max | Mean | SD |
| --- | --- | --- | --- | --- | --- | --- | --- | --- | --- |
|  | 1,142 | 929772 | -1.000 | -0.016 | < 0.001 | 0.040 | 1.000 | 0.009 | 0.150 |
| D’ | 11,418 | 930525 | -1.000 | -0.037 | < 0.001 | 0.028 | 1.000 | -0.004 | 0.154 |
|  | 22,836 | 931420 | -1.000 | -0.058 | < 0.001 | 0.017 | 1.000 | -0.019 | 0.162 |
|  | 1,142 | 1010904 | -1.000 | -0.007 | < 0.001 | 0.007 | 0.525 | < 0.001 | 0.037 |
| r^2^ | 11,418 | 1011611 | -1.000 | -0.008 | < 0.001 | 0.006 | 0.445 | -0.002 | 0.036 |
|  | 22,836 | 1012460 | -1.000 | -0.011 | -0.001 | 0.004 | 0.504 | -0.005 | 0.037 |

*Change in simulated LD from original HapMap sample LD for each pair of SNPs in Gene Region 1 (LD_simulated_ – LD_HapMap_).

**Sum of SNP pairs over all 100 replicates. The number of SNP pairs is not divisible by 100 because monoallelic SNPs were dropped from the LD calculations.

Table S6. Starting Locus Variation—Gene Region 1*

|  | Starting Locus | N** | Min | Q1 | Median | Q3 | Max | Mean | SD |
| --- | --- | --- | --- | --- | --- | --- | --- | --- | --- |
| D’ | random | 930525 | -1.000 | -0.037 | < 0.001 | 0.028 | 1.000 | -0.004 | 0.154 |
|  | 90770374 | 930343 | -1.000 | -0.074 | -0.004 | 0.065 | 1.000 | -0.005 | 0.187 |
|  | 90955029 | 930148 | -1.000 | -0.038 | < 0.001 | 0.028 | 1.000 | -0.005 | 0.156 |
|  | 91052395 | 930293 | -1.000 | -0.038 | < 0.001 | 0.029 | 1.000 | -0.003 | 0.155 |
| r^2^ | random | 1011611 | -1.000 | -0.008 | < 0.001 | 0.006 | 0.445 | -0.002 | 0.036 |
|  | 90770374 | 1011613 | -1.000 | -0.011 | -0.001 | 0.007 | 0.475 | -0.003 | 0.037 |
|  | 90955029 | 1011326 | -1.000 | -0.009 | < 0.001 | 0.005 | 0.578 | -0.003 | 0.036 |
|  | 91052395 | 1011471 | -1.000 | -0.009 | < 0.001 | 0.006 | 0.596 | -0.003 | 0.036 |

*Change in simulated LD from original HapMap sample LD for each pair of SNPs in Gene Region 1 (LD_simulated_ – LD_HapMap_).

**Sum of SNP pairs over all 100 replicates. The number of SNP pairs is not divisible by 100 because monoallelic SNPs were dropped from the LD calculations.

Table S7. Hapgen Recombination Rate Variation—Gene Region 1

|  | Recombination Rate Weight | N | Min | Q1 | Median | Q3 | Max | Mean | SD |
| --- | --- | --- | --- | --- | --- | --- | --- | --- | --- |
|  | 0.1 | 932088 | -1.000 | -0.011 | < 0.001 | 0.043 | 1.000 | 0.012 | 0.148 |
| D’ | 1 | 930525 | -1.000 | -0.037 | < 0.001 | 0.028 | 1.000 | -0.004 | 0.154 |
|  | 10 | 931476 | -1.000 | -0.197 | -0.063 | < 0.001 | 1.000 | -0.114 | 0.221 |
|  | 0.1 | 1013312 | -1.000 | -0.006 | < 0.001 | 0.008 | 0.614 | 0.001 | 0.035 |
| r^2^ | 1 | 1011611 | -1.000 | -0.008 | < 0.001 | 0.006 | 0.445 | -0.002 | 0.036 |
|  | 10 | 1012746 | -1.000 | -0.031 | -0.006 | < 0.001 | 0.440 | -0.027 | 0.057 |

*Change in simulated LD from original HapMap sample LD for each pair of SNPs in Gene Region 1 (LD_simulated_ – LD_HapMap_).

**Sum of SNP pairs over all 100 replicates. The number of SNP pairs is not divisible by 100 because monoallelic SNPs were dropped from the LD calculations.

Table S8. Resampling Recombination Rate Variation—Gene Region 1

|  | Recombination Rate Weight | N | Min | Q1 | Median | Q3 | Max | Mean | SD |
| --- | --- | --- | --- | --- | --- | --- | --- | --- | --- |
|  | 0.1 | 1015158 | -0.602 | -0.007 | < 0.001 | 0.010 | 1.000 | 0.003 | 0.059 |
| D’ | 1 | 1015300 | -0.991 | -0.006 | < 0.001 | 0.010 | 0.993 | 0.003 | 0.058 |
|  | 10 | 1015300 | -1.000 | -0.016 | < 0.001 | 0.006 | 1.000 | -0.002 | 0.063 |
|  | 0.1 | 1015158 | -0.336 | -0.003 | < 0.001 | 0.003 | 0.262 | < 0.001 | 0.014 |
| r^2^ | 1 | 1015300 | -0.254 | -0.003 | < 0.001 | 0.003 | 0.259 | < 0.001 | 0.014 |
|  | 10 | 1015300 | -0.210 | -0.004 | < 0.001 | 0.003 | 0.235 | -0.001 | 0.015 |

*Change in simulated LD from original HapMap sample LD for each pair of SNPs in Gene Region 1 (LD_simulated_ – LD_HapMap_).

**Sum of SNP pairs over all 100 replicates. The number of SNP pairs is not divisible by 100 because monoallelic SNPs were dropped from the LD calculations.
